# Supplementary material for: Inertial and viscous flywheel sensing of nanoparticles
Source: Nat Commun. 2021 Aug 24;12:5099. doi: 10.1038/s41467-021-25266-3 (PMC8385060; doi:10.1038/s41467-021-25266-3)
Supplement: Supplementary file 1 — Supplementary Info [file 41467_2021_25266_MOESM1_ESM.pdf]

Supplementary information

Inertial and viscous flywheel sensing of nanoparticles

Georgios Katsikis,[1,6] Jesse F. Collis,[2,6] Scott M. Knudsen,[1] Vincent Agache,[1,3] John E. Sader,[2*] Scott R. Manalis[1,4,5*]

[1]Koch Institute for Integrative Cancer Research, Massachusetts Institute of Technology, Cambridge, MA 02139 USA.
[2]ARC Centre of Excellence in Exciton Science, School of Mathematics and Statistics, The University of Melbourne, VIC 3010, Australia.
[3]Université Grenoble Alpes, CEA, LETI, 38000, Grenoble France.
[4]Department of Biological Engineering, Massachusetts Institute of Technology, Cambridge, MA, 02139 USA.
[5]Department of Mechanical Engineering, Massachusetts Institute of Technology, Cambridge, MA, 02139 USA.
[6]These authors contributed equally: Georgios Katsikis, Jesse F. Collis.
[*]email: jsader@unimelb.edu.au; srm@mit.edu.

Supplementary Figures

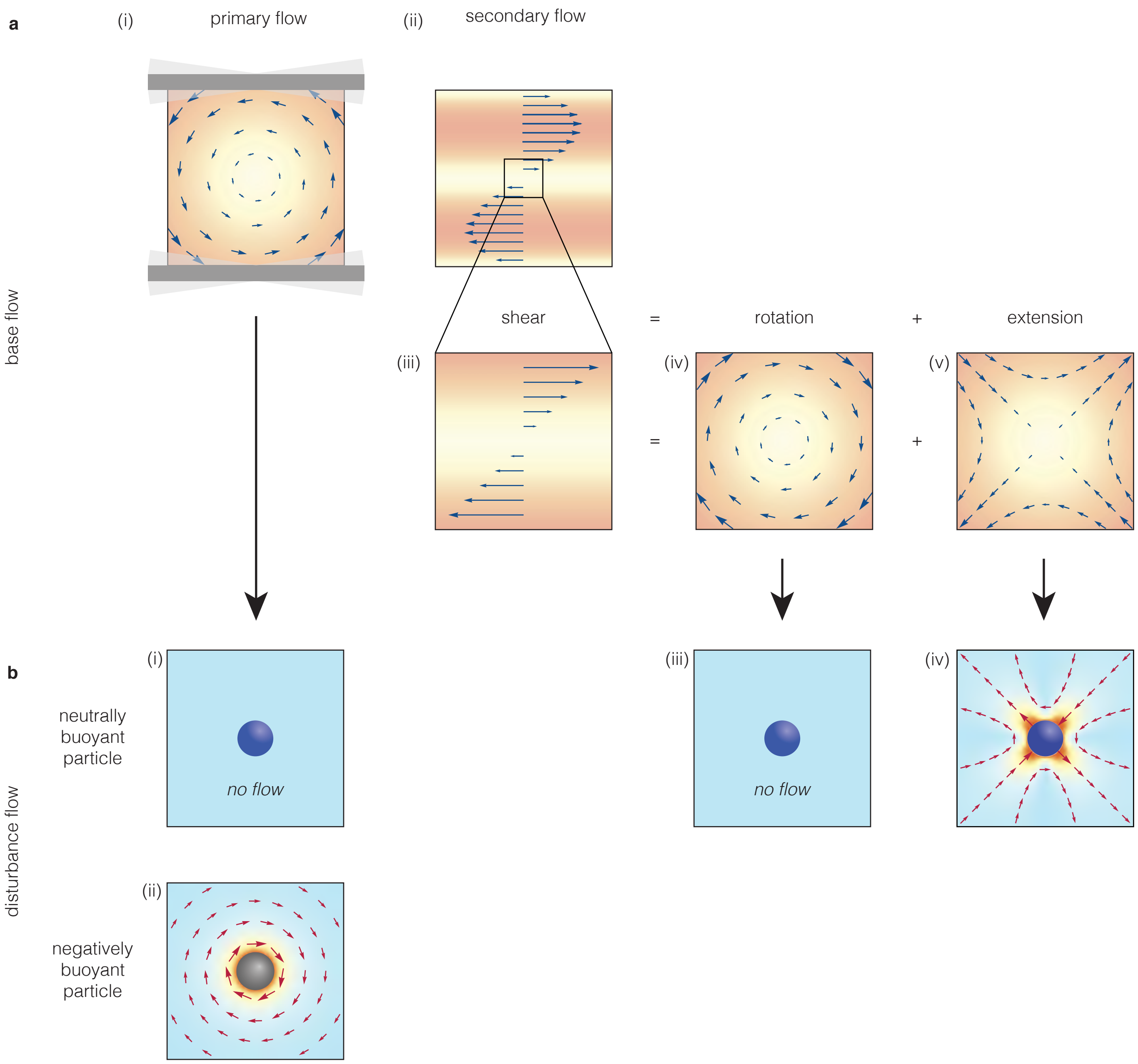

Supplementary Fig. 1 | Illustration of the flow within a rotating microchannel at low Reynolds number. a, Flow fields generated within the rotating microchannel in the absence of a suspended particle. (i) The flow is a rigid body rotation in the limit of zero inertia. (ii) Infinitesimal inertia causes the fluid to lag its primary flow counterpart. This produces a secondary, non-linear, shear flow. (iii) As the theory assumes the suspended particle is much smaller than the channel height ($a \ll H$), this non-linear shear flow is approximated as linear over the length scale of the particle. (iv, v) A pure (linear) shear flow is decomposable into a pure rotation and a pure extension, i.e., $z\hat{\mathbf{x}} = (z\hat{\mathbf{x}} - x\hat{\mathbf{z}})/2 + (z\hat{\mathbf{x}} + x\hat{\mathbf{z}})/2$. **b**, Flow due to a particle suspended in the base flows in (a). (i) A neutrally buoyant particle rotates with the same angular velocity as the base flow and therefore produces no disturbance flow. (ii) A negatively buoyant particle produces a rotational disturbance flow and is discussed further in the caption of Fig. 1. (iii) The neutrally buoyant particle does not react to the rotational component of the secondary shear flow for the same reason as (i). (iv) It does, however, react to the shear flow's extensional component, producing a quadrupole disturbance flow (iv), similar to the case for high inertia in Fig. 1. The difference between (iv) here and Fig. 1c-ii is that the flow around the particle is dominated by viscosity in the former and inertia in the latter.

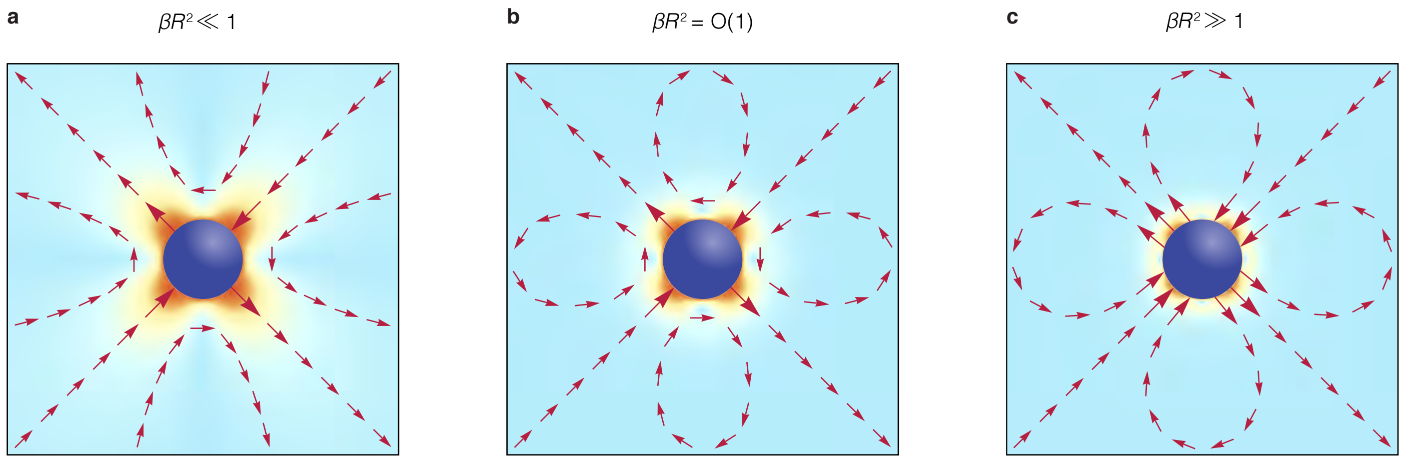

Supplementary Fig. 2 | Quadrupole disturbance flows (i.e., particle reaction to an extensional base flow) for increasing particle/viscous penetration depth ratio. a, For $\beta R^2 \ll 1$, the disturbance flow generated by the particle is viscous everywhere and decays at a rate of $1/r^2$ where r is the non-dimensional distance from the particle surface. The streamlines are always open, i.e., there are no recirculation zones. This regime results in the strongest disturbance flow of the three results presented here, and hence also the largest magnitude of α_v. **b**, For $\beta R^2 = O(1)$, the disturbance flow is viscous dominated from the particle surface to a distance of $O(1)$ from the particle surface; the flow decays at a rate of $1/r^2$ in this region. Outside this region, the flow is primarily inviscid, where it decays at a rate of $1/r^4$. This results in a weaker disturbance flow than (a), and hence a reduction to the magnitude of α_v. **c**, For $\beta R^2 \gg 1$, the disturbance flow is primarily inviscid and so decays at a rate of $1/r^4$ from the particle surface. This results in a weaker disturbance flow than (b) and hence the smallest magnitude of α_v for the three results presented here.

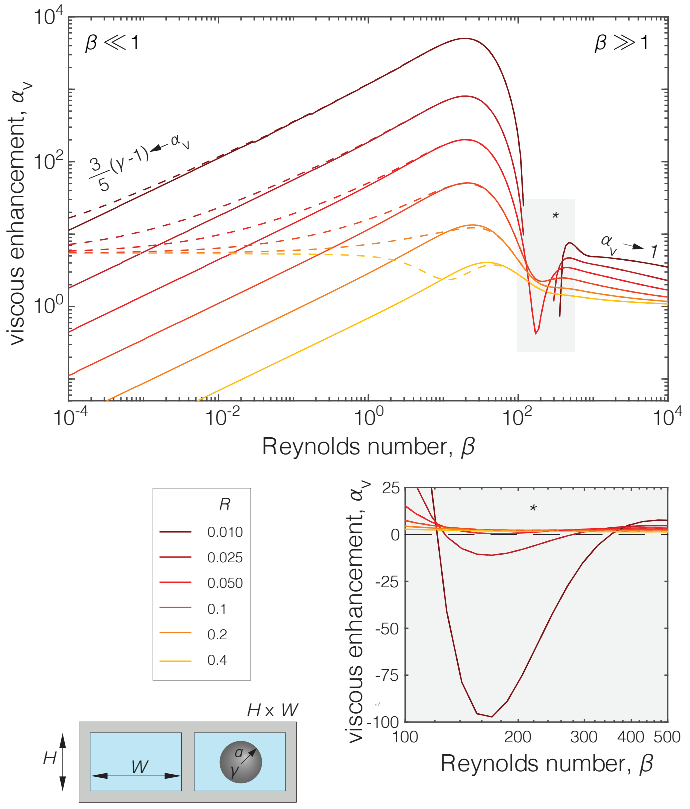

Supplementary Fig. 3 | Viscous enhancement factor, α_v, vs Reynolds number β for varying ratios of particle radius to channel height ($R = a/H$). Order of curves matches order of values in legend from top to bottom. Assuming that the particle is located at the center of the channel ($z = 0$), for most Reynolds numbers, decreasing particle size, and hence increasing the effects of viscosity in the vicinity of the particle (Supplementary Fig. 2), increases the magnitude of α_v. At intermediate β, the complex interplay between the particle size, and the viscous boundary layers in the vicinity of the particle and the channel walls, results in a sensitive dependence of α_v on R, even reversing the sign of α_v (gray inset with asterisk). The ratio of particle density over fluid density, $\gamma = \rho_p/\rho$, is shown with continuous lines for $\gamma = 1$ and dashed lines for $\gamma = 10$. The curve for $R = 0.1$ and $\gamma = 1$ is identical to the corresponding curve in Fig. 2.

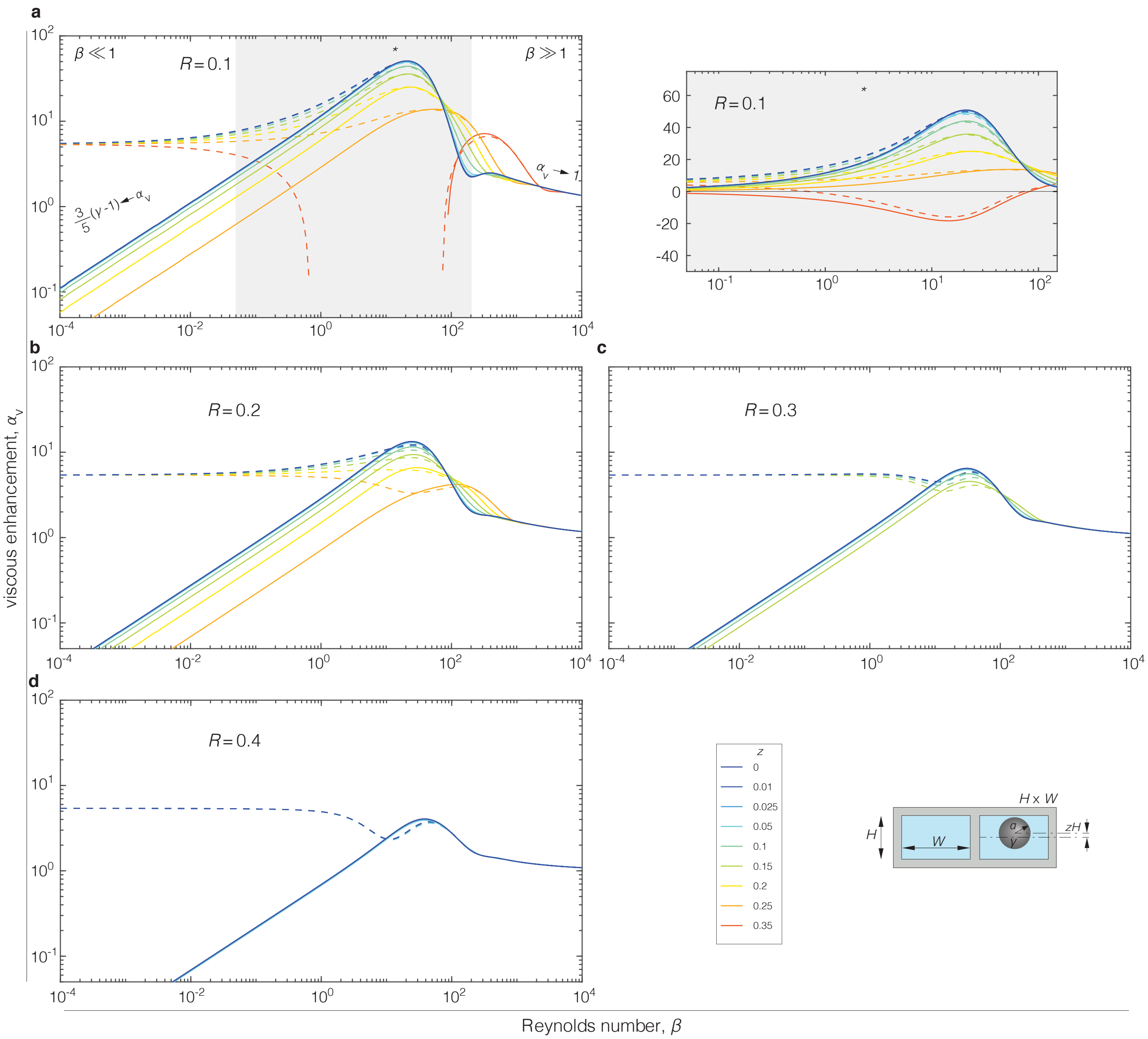

Supplementary Fig. 4 | Viscous enhancement factor, α_v, vs Reynolds number, β, for varying z-position. a-d, Four different radii, $R = 0.1, 0.2, 0.3, 0.4$, respectively shown in **a-d**, for z-positions in the range $0 - 0.35$. Order of curves matches order of values in legend from top to bottom. Radii are defined as $R = a/H$ where a is the dimensional radius of the particle. For $R = 0.1$, at intermediate β, α_v reverses sign as shown in the log-linear plot for the gray inset (denoted by asterisk) in **a**. Only positive z is shown here as α_v is symmetric in z. The limits $\beta \ll 1$ and $\beta \gg 1$, and the corresponding asymptotic formulae, are shown only in (a) but apply to all panels. As a particle cannot cross over a wall, the maximum z-position is given by $z_{max} = 0.5 - R$, hence the decreasing number of curves from (b)-(d). The ratio of particle density over fluid density $\gamma = \rho_p/\rho$ is shown with continuous lines for $\gamma = 1$ and dashed lines for $\gamma = 10$. The curve for $R = 0.1$ and $z = 0$ is identical to the corresponding curve in Fig. 2. Note that, while the theory is derived in the limit $R \ll 1$, we find good agreement between theory and experiment up to the experimental maximum of $R = 0.4375$.

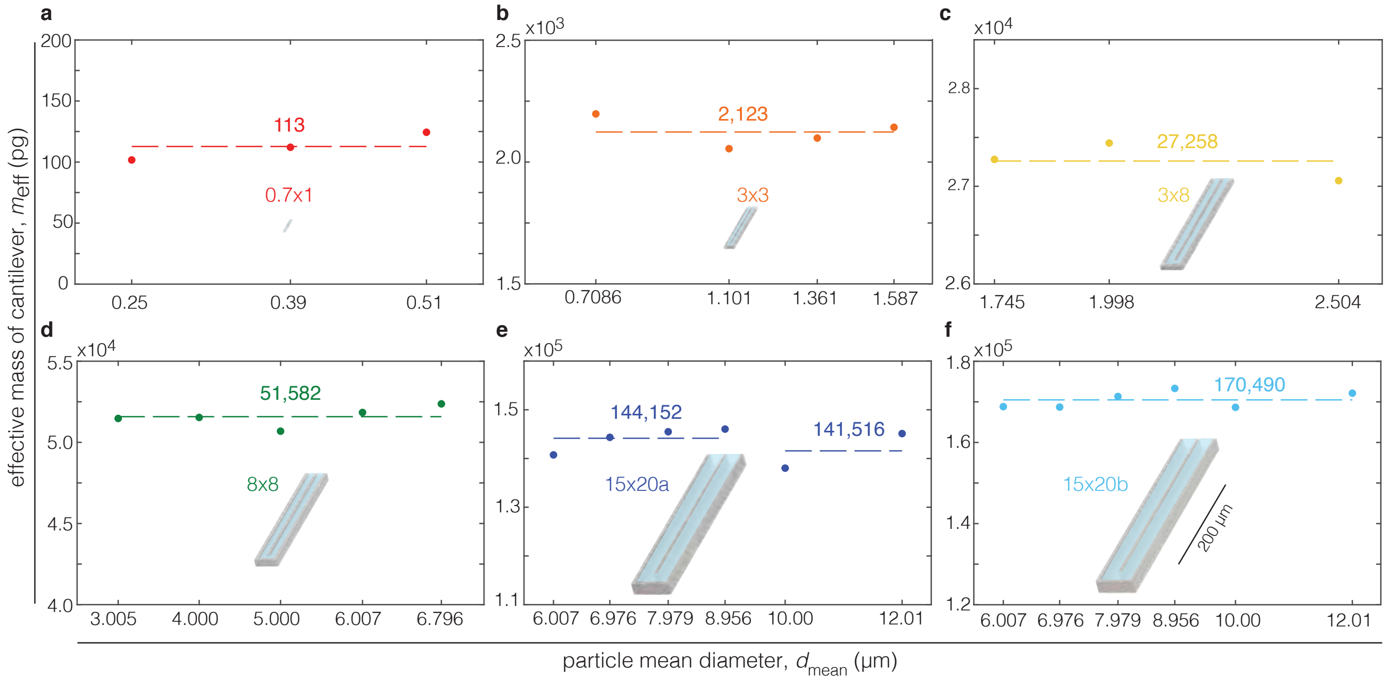

Supplementary Fig. 5 | Calculation of the effective mass, m_{eff} for each cantilever used in this study. **a-f**, Code names and colors are the same as in Figs. 2, 4. For each cantilever, m_{eff} is calculated by applying Supplementary Eq. (20a) at the antinode position (Fig. 3b) where Δf^{anti} and f are measured experimentally. The buoyant mass, m_{b}, is calculated as $m_{\text{b}} = (\rho_{\text{p}} - \rho)\pi d_{\text{mean}}^3/6$ where d_{mean} is the mean diameter of a given monodisperse solution of polystyrene calibration particles and $\rho_{\text{p}} = 1{,}050\ \text{kg/m}^3$ is their density (Supplementary Table 2). The nominal diameter d_{nom} was not available for the particles in the device 0.7×1.0, so d_{mean} was used for calibration. The dashed lines represent the mean value of m_{eff} calculated from solutions of different d_{nom} in each cantilever. Two cantilevers of type 15×20a were used.

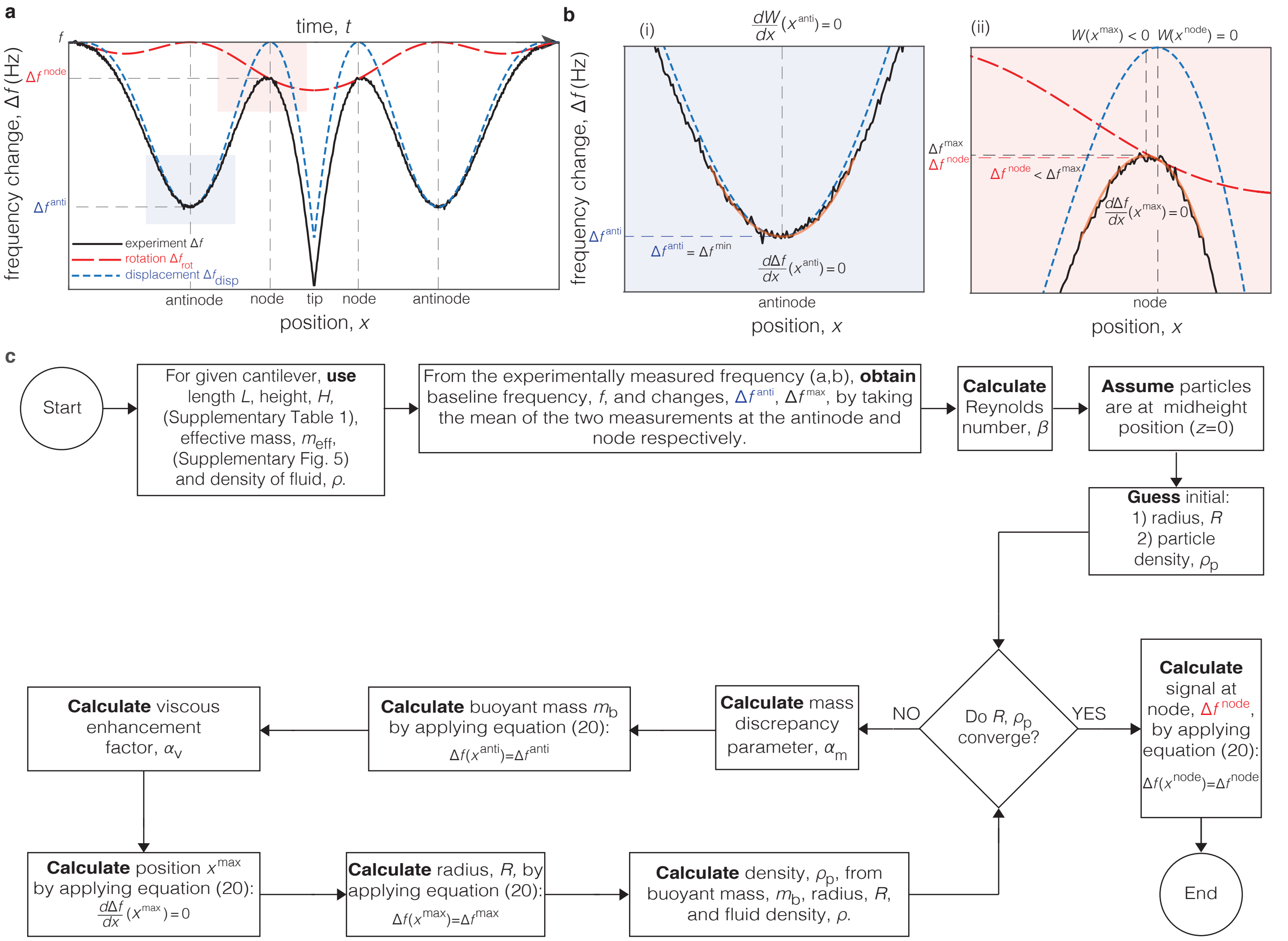

Supplementary Fig. 6 | Extraction of signals from measured resonant frequency changes and corresponding iteration algorithm for calculating particle properties. a, Frequency change, Δf, induced by a particle vs x-position along the length of the cantilever. The experimental signal, Δf (black), consists of two signals: Δf_{disp} due to displacement (blue) and Δf_{rot} due to rotation (red), similar to Fig. 3b but shown here for the double measurement, i.e., the particle transit down each side of the cantilever (Fig. 3a). **b**, Frequency change, Δf, induced in the vicinity of the (i) antinode and (ii) node with background colors corresponding to areas in (a). The orange curves represent the smoothed curves for extracting the local $\Delta f^{\mathrm{min}} = \Delta f^{\mathrm{anti}}$ (i) and (ii) Δf^{max}. Note that Δf^{max} at the x-position of the local maximum in the black curve of (ii) does not exactly correspond node signal Δf^{node} with $\Delta f^{\mathrm{node}} < \Delta f^{\mathrm{max}}$ and $x^{\mathrm{node}} > x^{\mathrm{max}}$. **c**, Flow chart of the iterative algorithm used to calculate the particle properties.

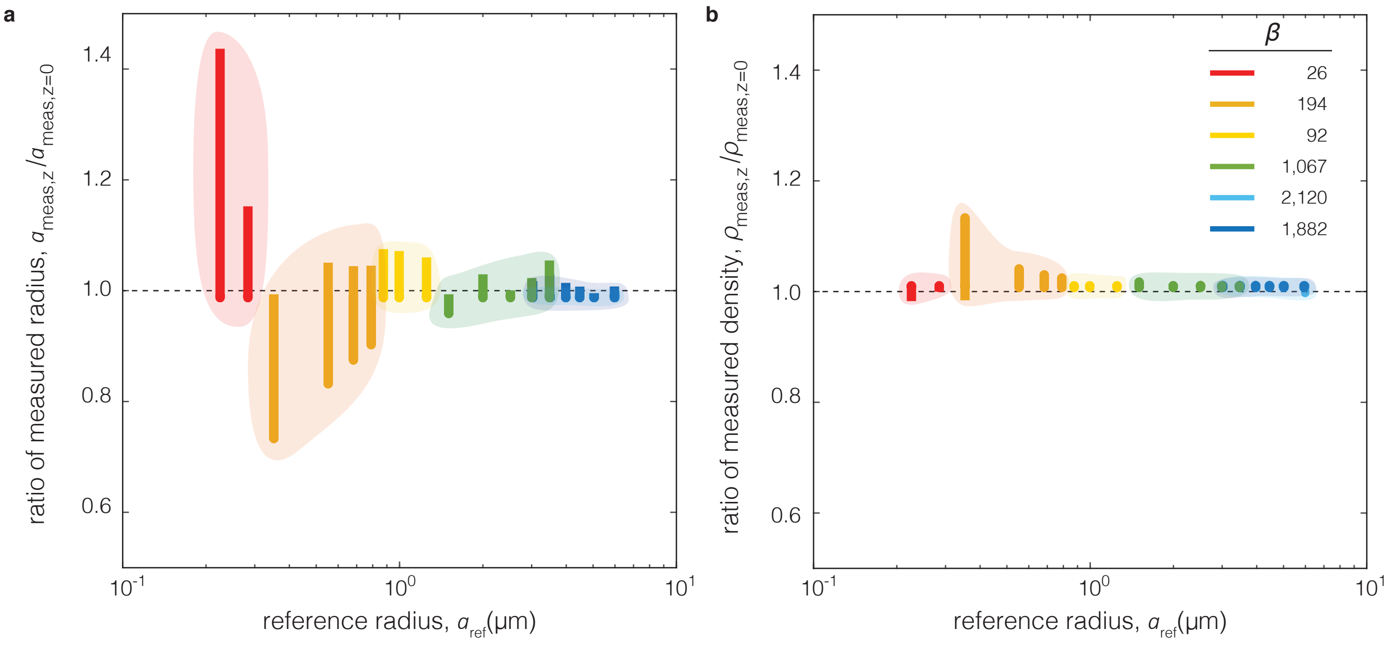

Supplementary Fig. 7 | Uncertainty in the measured diameter and density based on z-position of the particle. a,b, The variables $a_{z=0}$ and $\rho_{z=0}$ specify the measured radius and density assuming the particle is located at $z = 0$. a_z and ρ_z specify the measured radius and density assuming the particle to be located anywhere in the range $0 \leq z \leq z_{\max}$ where $z_{\max}$ is the closest location to the wall that the particle is able to occupy (Supplementary Fig. 4). Colors correspond to those in Fig. 4 and the oscillatory Reynolds numbers β are those given in Supplementary Table 1; islands from left to right correspond to β values in the legend top to bottom with the last two islands nearly completely overlapping. The bands give 95% confidence intervals across all meaurements. In general, the smaller both β and R become, the larger the error due to uncertainty in z-position; see Supplementary Fig. 4 where the difference between the top and bottom curves corresponds to the degree of uncertainty. For the smallest particle in the smallest device, α_v reverses sign near to the walls and so by assuming a z-position in this region we cannot fit a diameter and density to the data.

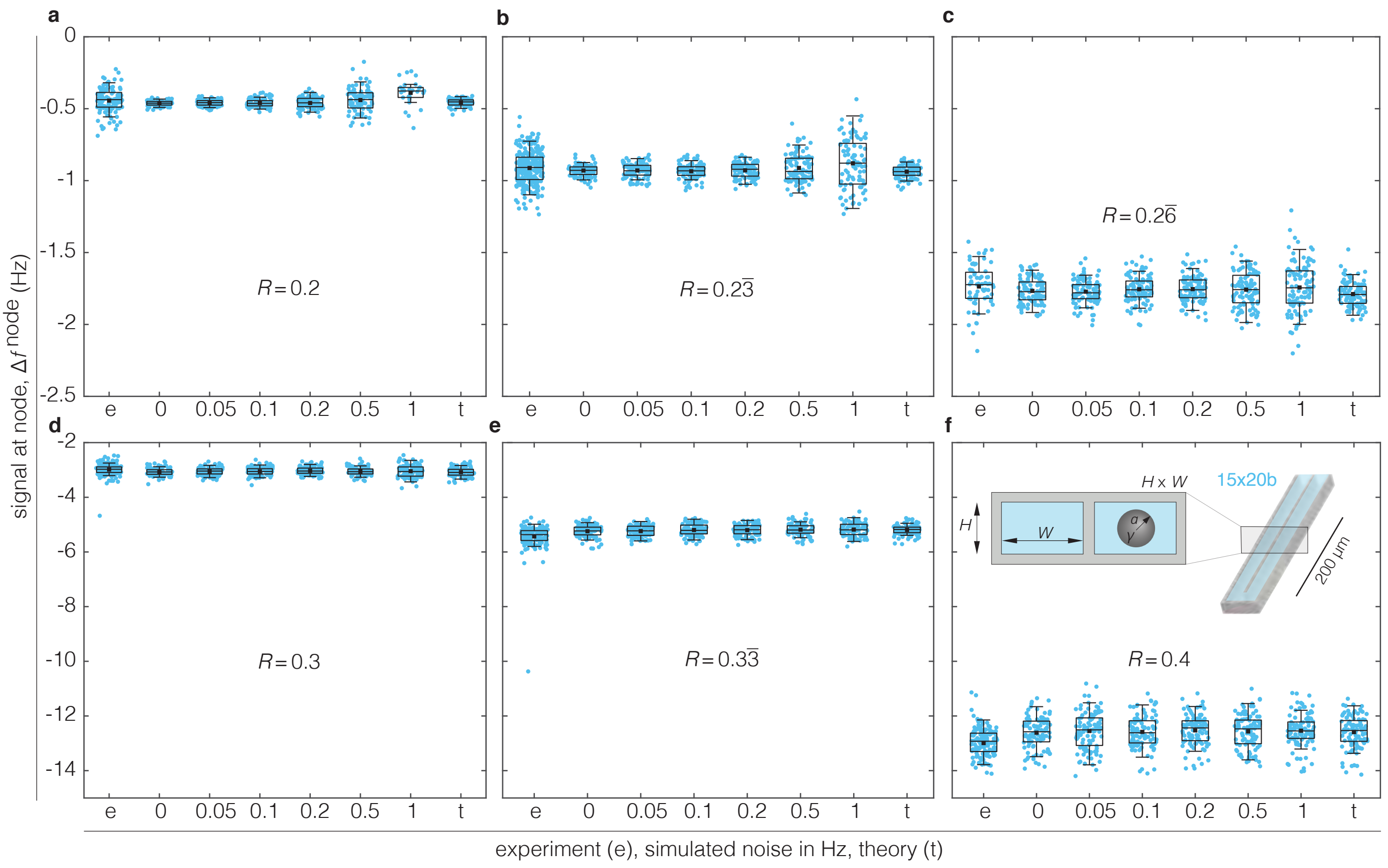

Supplementary Fig. 8 | Comparison of the node signal from experiments with Monte-Carlo simulations at varying noise levels. **a-f**, Signal at node position, Δf^{node}, (Fig. 3b) for particles of nominal diameter $d_{\text{nom}} = 6 - 12$ µm (corresponding to $R = 0.2 - 0.4$) in the 15×20b device (Fig.4, 15×20b). Radii are defined as $R = a/H$ where a is the dimensional radius of the particle. The inset in **f** shows the cross section of the flow channel with area $H \times W$. Noise simulations consist of theoretically calculated Δf^{node} using Eq. (1) with added white Gaussian noise of standard deviation $\sigma = 0, 0.05, 0.1, 0.2, 1$ Hz with $n = 100$ individually simulated points. Frequency changes, Δf, from experiments (symbol e) and noise simulations (horizontal axis numbers have the units Hz) are processed with the same algorithm for extracting Δf^{node} from experiments (Supplementary Fig. 6). Signals of Δf^{node} from either experiments or simulations are rejected if the signal-to-noise ratio is low (for example in (a), 1 Hz). The (noiseless) theory (symbol t) is calculated directly from Eq. (1). The general trend observed here is that noise dominates the signal for small particles ($d_{\text{nom}} \leq 7$ µm or $R \leq 0.2\overline{6}$), and polydispersity dominates the signal for larger particles. The central mark indicates the median. The black squares the mean. The bottom and top edges of the boxes indicate the 25th and 75th percentiles, respectively. The bottom and top whiskers indicate the 5th and 95th percentiles. All percentiles are defined by assuming points follow normal distribution.

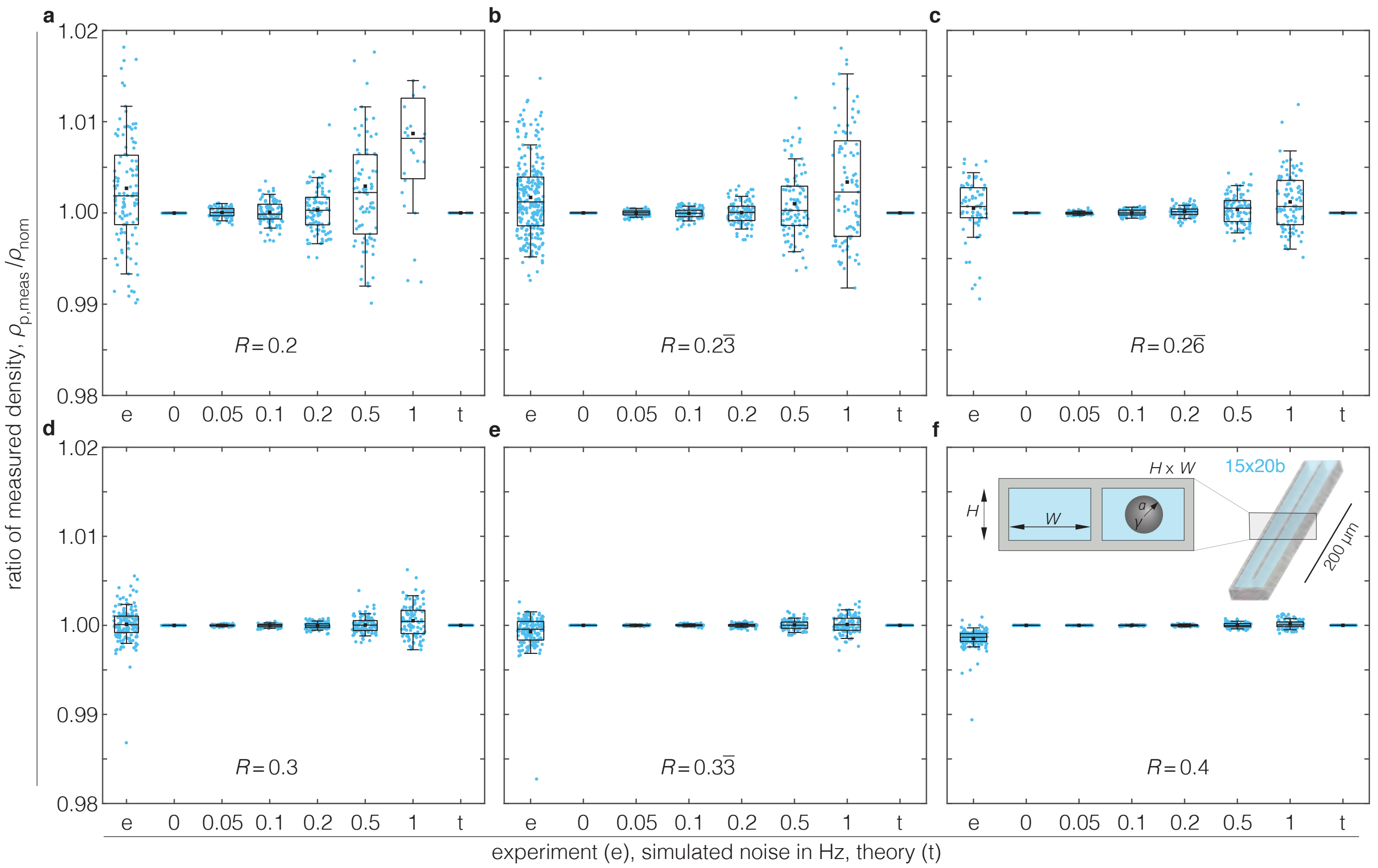

Supplementary Fig. 9 | Comparison of microparticle density calculated from experiments, with Monte-Carlo simulations varying the noise level. **a**-**f**, Measured density ratios $\rho_{\mathrm{p,meas}}/\rho_{\mathrm{nom}}$ of particles of nominal diameter $d_{\mathrm{nom}} = 6 - 12$ μm (corresponding to $R = 0.2 - 0.4$) in the 15×20b device (Fig.4, 15×20b). The data, insets, numbers, and box plot symbols are the same as in Supplementary Fig. 8. As R increases, $\rho_{\mathrm{p,meas}}/\rho_{\mathrm{nom}} \to 1$ due to a higher signal to noise ratio. However, for the maximum diameter $d_{\mathrm{nom}} = 12$ μm ($R = 0.4$), there is a deviation of the experimental data (symbol e) from $\rho_{\mathrm{p,meas}}/\rho_{\mathrm{nom}} = 1$ which is likely due to the effects of finite particle size. Boxplots have similar notation as in Supplementary Fig. 7.

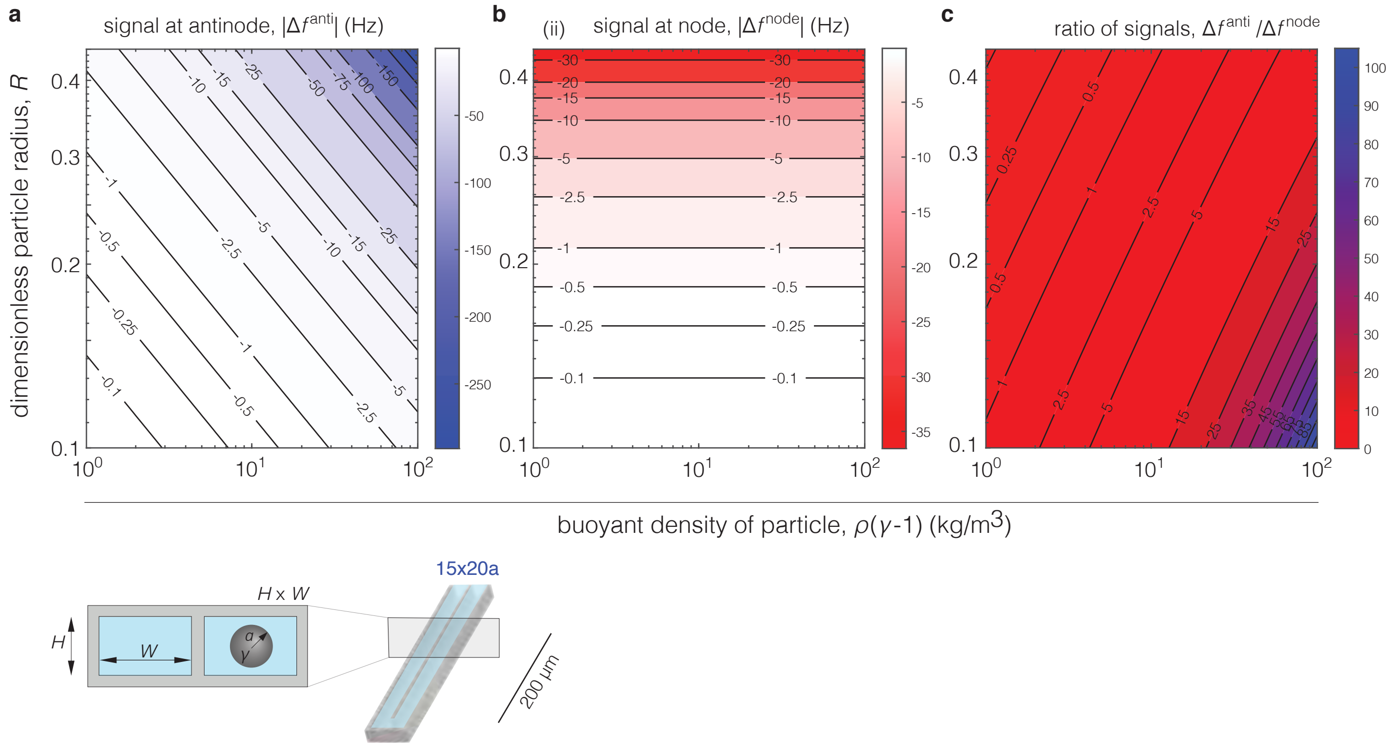

Supplementary Fig. 10 | Variation of antinode and node signals across a range of particle buoyant densities and sizes relative to cantilever height. Theoretical calculations of, **a**, Δf^{anti}, **b**, Δf^{node} and, **c**, ratio $\Delta f^{\mathrm{anti}}/\Delta f^{\mathrm{node}}$ vs dimensionless radius $R = a/H$ for the 15×20a cantilever (bottom schematic) with the highest, Reynolds number, β, tested experimentally (Figs. 2, 4). Ratio of the particle density to the fluid density is $\gamma = \rho_{\mathrm{p}}/\rho$, thus the buoyant density is $\rho(\gamma - 1)$. For most of this range we observed that the antinode signal is greater than the node signal $\Delta f^{\mathrm{anti}}/\Delta f^{\mathrm{node}} > 1$.

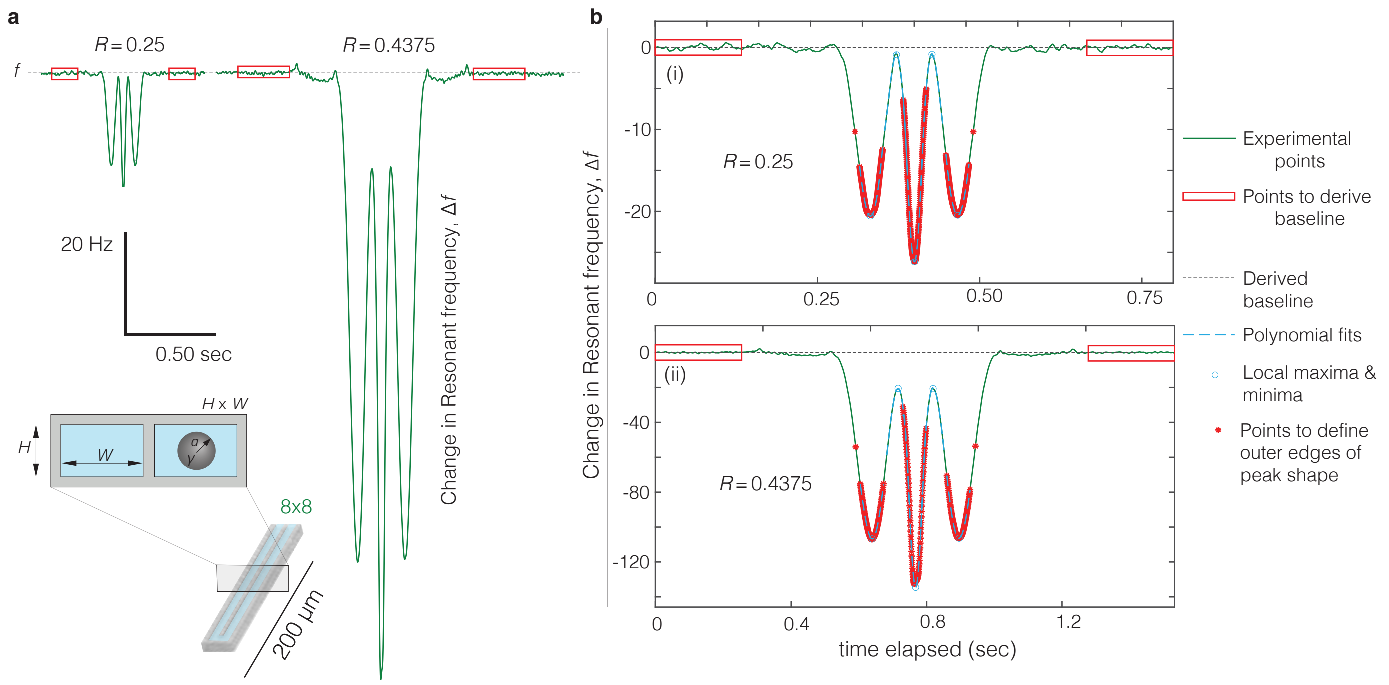

Supplementary Fig. 11 | Sensitivity of the calculation of resonant frequency changes to the determination of the baseline frequency. **a**, Two examples of experimentally measured resonant frequencies, Δf, in the 8×8 device for particles of $d_{\mathrm{nom}} = 4, 7$ µm. (corresponding to $R = 0.25, 0.4375$) in the 8×8 device (Fig. 4, 8×8). f denotes the frequency of the baseline before and after the passage of the particle into the cantilever. The bottom schematic shows the cross section of the flow channel with area $H \times W$. The red rectangles define the regions before and after the passage of the particle in the cantilever, where a linear fit is done to establish the baseline frequency, f. In the case of $d_{\mathrm{nom}} = 7$ µm, where the particle size is comparable to H, the particle perturbs the baseline frequency before entering the cantilever, introducing an error into the calculation of f. In addition, the theory is derived in the limit $R \ll 1$, adding additional error to the interpretation of these measurements. **b**, Calculation of resonant frequency changes at the node and antinode, Δf^{node} and Δf^{anti} respectively, from the frequency change, Δf, for (i) $d_{\mathrm{nom}} = 4$ µm and (ii) $d_{\mathrm{nom}} = 7$ µm. The user-defined extraction of f introduces an uncertainty in the calculation of Δf, which affects Δf^{node} more than Δf^{anti} due to the fact that, generally, the former has lower magnitude than the latter (Fig. 4a, Supplementary Fig. 12).

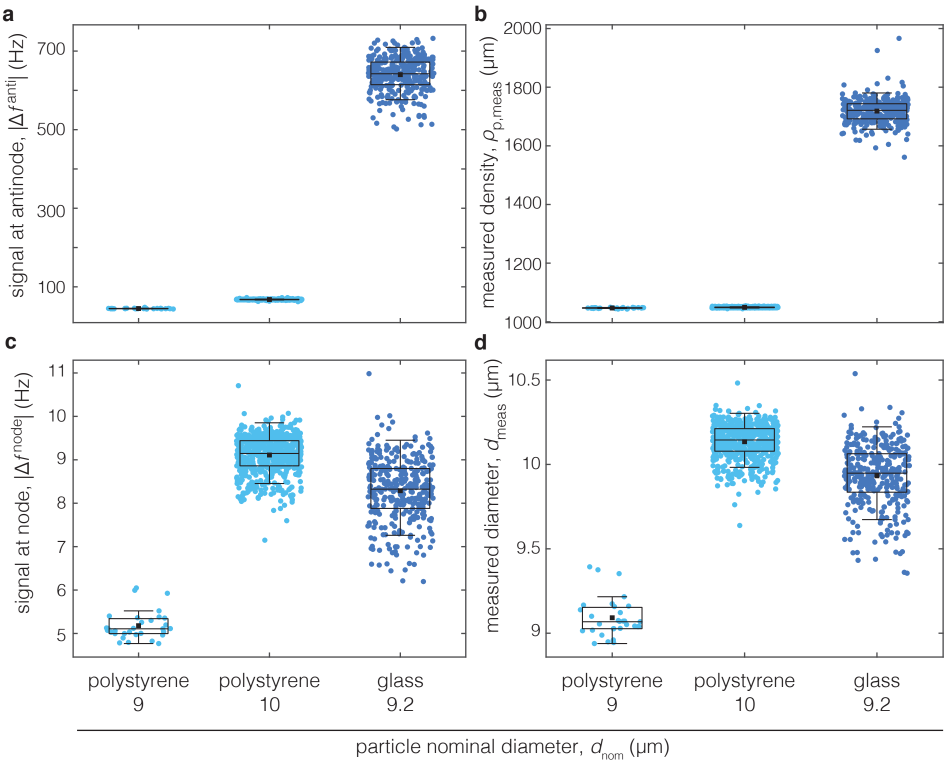

Supplementary Fig. 12 | Using the node signal to measure the density of particles of different composition. **a,c** Measurements of the signals, Δf^{node} and Δf^{anti}, ($\Delta f^{\mathrm{node}}, \Delta f^{\mathrm{anti}} < 0$) at the node and antinode positions respectively (Fig. 3b) for three different particle samples in water suspensions: (1) polystyrene particles of nominal diameter $d_{\mathrm{nom}} = 9, 10$ µm (light blue) with and (2) $\rho_{\mathrm{p,nom}} = 1{,}050$ kg/m^3 (same data as in Fig. 4), and (3) glass microparticles of $d_{\mathrm{nom}} = 9.2$ µm and $\rho_{\mathrm{p,nom}} = 2{,}000$ kg/m^3. The total number of measurements for the three samples are respectively $n = 31, 590, 334$. As opposed to the polystyrene particles, the glass particles are not calibration standards and were expected to deviate from the specifications of the manufacturer, specifically, the density of these particles is not well characterized. **b,d** Measured diameter, d_{meas}, and density, ρ_{meas}, for three samples calculated using Δf^{node} and Δf^{anti}. As predicted by the theory, the glass particles have a similar node signal, Δf^{node}, as polystyrene particles of similar volume but an order of magnitude higher antinode signal, Δf^{anti}, due to the corresponding difference in buoyant density $\Delta\rho_{\mathrm{b}} = \rho_{\mathrm{p}} - \rho$. Experiments were performed in the 15×20a devices. Boxplots have similar notation as in Supplementary Fig. 7.

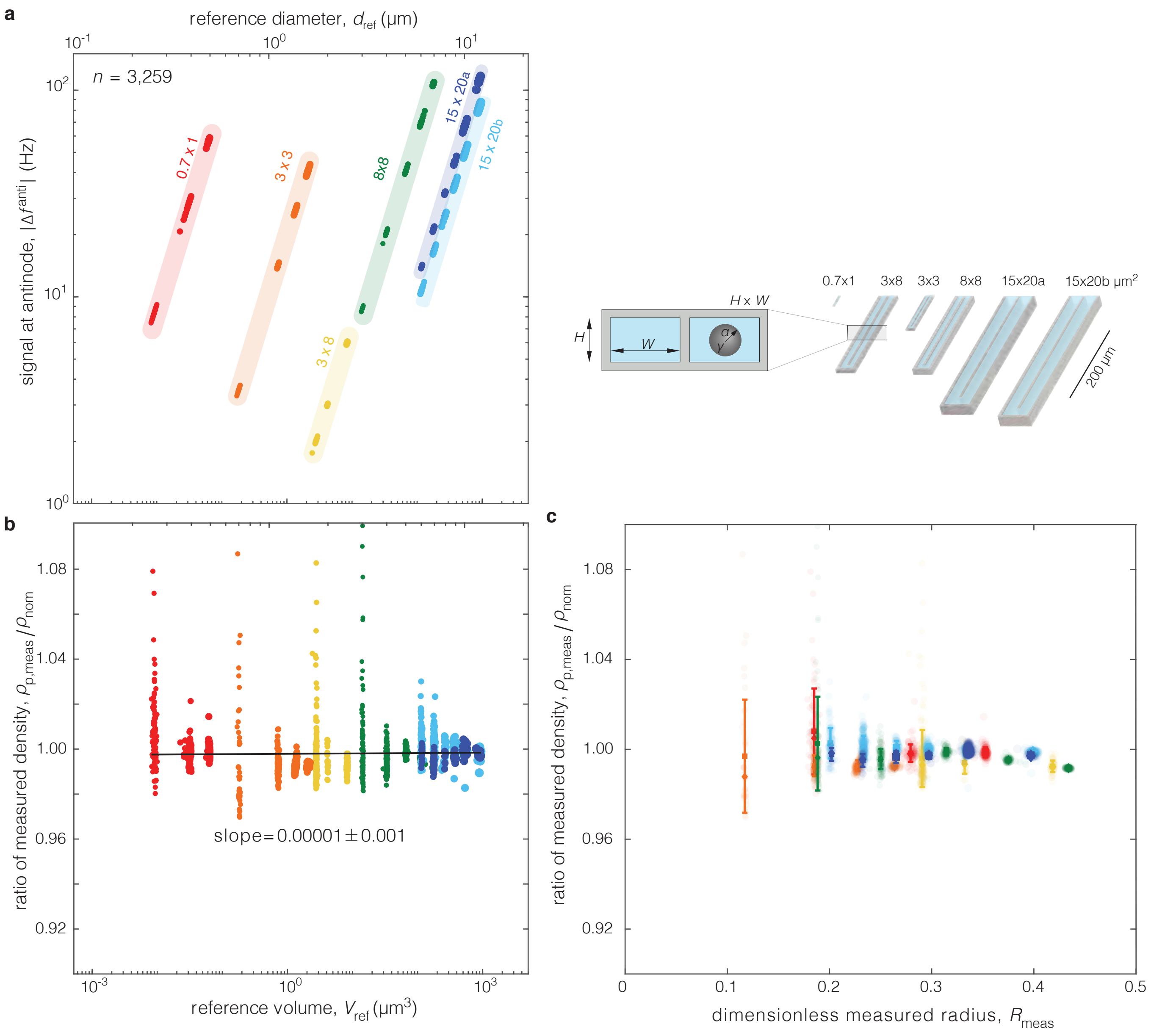

Supplementary Fig. 13 | Measuring particle density from a single measurement. **a**, Measurements of the frequency change signal Δf^{anti} at the antinode position (Fig. 3b) vs reference particle diameter, d_{ref}, (top horizontal axis) and reference particle volume, V_{ref}, (bottom horizontal axis) for the same experiments shown in Fig. 4 using identical notation with colors and symbols. Note that in the present experiments, $\Delta f^{\text{anti}} < 0$. Δf^{anti} is depicted without noise because it is plotted vs $d_{\text{ref}}/V_{\text{ref}}$ which are calculated directly from Δf^{anti} using Eq. (20a). **b**, Ratio of measured density $\rho_{\text{p,meas}}/\rho_{\text{nom}}$ vs d_{ref} (top horizontal axis) V_{ref} (bottom horizontal axis), where ρ_{meas} is calculated from experimental signals of Δf^{node} (Fig. 4a**)** and Δf^{anti} using the viscous enhancement factor, α_{v} (Fig. 2), and effective mass, m_{eff} (Supplementary Fig. 5), using an iterative algorithm (Supplementary Fig. 6). The linear regression of V_{meas} is done vs V_{ref} for a 99.9% confidence interval. **c**, Ratio of measured density $\rho_{\text{p,meas}}/\rho_{\text{nom}}$ vs $R_{\text{ref}} = a_{\text{ref}}/H$, where a_{ref} is the reference radius of the particle; see schematic in center-right. For every dataset of given R_{ref}, squares denote mean, diamonds median, and error bars are standard deviation. For all devices, decreasing R_{meas} increases error. Color notation similar to (a, b**)** but individual data points are shown transparent for illustration purposes.

Supplementary Tables

Schematic of SNR/SMR cantilever devices

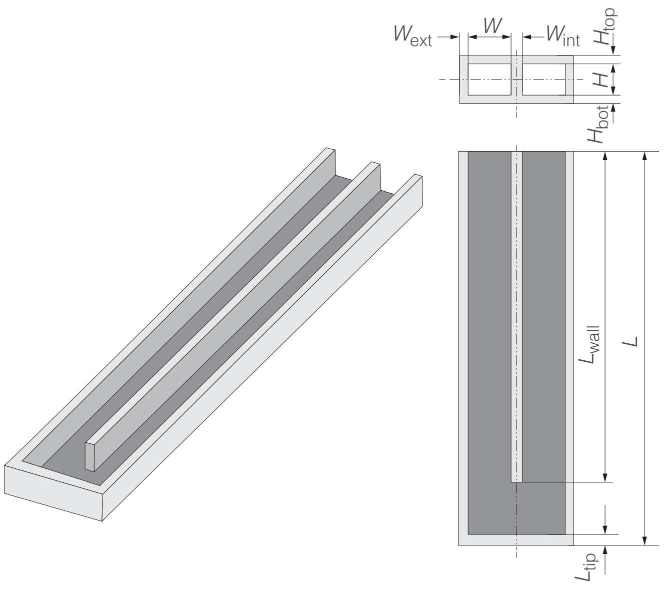

Dimensions (µm)

name	0.7x1	3x3	3x8	8x8	15x20a	15x20b
H	0.7	3	3	8	15	15
W	1.0	3	8	8	20	20
L	32.5	100	210	210	321	355
H_{bot}	0.2	1	2	2	2	2
H_{top}	0.2	1	2	2	2	2
W_{ext}	1.0	2	6	6	6	6
W_{int}	0.5	1.5	5	5	5	5
L_{wall}	31.0	98	196	196	295	329
L_{tip}	0.5	1	6	6	6	6
Baseline frequency f (MHz), Reynolds number β, Quality factor Q						
f	7.54	3.06	1.46	2.37	1.34	1.19
β	26	194	92	1,067	2,120	1,882
Q	3,000	1,800	4,000	1,600	800	1,000

Supplementary Table 1 | Characteristics of the cantilevers used in this study. Schematic of a cantilever with its geometric dimensions. The cantilever in the perspective view is shown without its top lid for clarity; the cantilever has a top lid with thickness $H_{\mathbf{top}}$ as shown in the cross-section view of the flow channel. All cantilevers have the same geometrical features. The table gives the dimensions of each cantilever and their corresponding baseline frequency, f, Reynolds number, β, and quality factor, Q, when operated in 2nd resonant mode while filled with water (density and shear viscosity of the water are 997 kg/m^3 and 0.89 mPa s respectively). The bolded dimensions (H, L, f and β) are used directly in Eq. (1) to calculate the change in resonant frequency, Δf.

nominal diameter d_{nom} (μm)	0.25	0.39	0.51	0.7	1.1	1.3	1.6	1.8	2	2.5
diameter d_{mean} (mean±error) (μm)	not available			0.7086±0.0235	1.101±0.017	1.361±0.015	1.587±0.025	1.745±0.025	1.998±0.022	2.504±0.025
size distribution (μm)				-	0.012	0.021	0.021	0.019	0.020	0.025
coefficient of variation, CV (%)	5 to 10			3	1.1	1.5	1.3	1.1	1.0	1.0
nominal density ρ_{nom} (g/cm^3)	1.05 (polystyrene)									
catalog number	PS02N	PS02N	PS03N	NT12N	4011A	4013A	4016A	4018A	4202A	4025A
lot number	7307	6703	5970	7141	44653	44330	35209	35306	34646	35261
vendor	Bangs Laboratories				Thermo Scientific Duke Standards					

nominal diameter d_{nom} (μm)	3	4	5	6	7	8	9	10	12
diameter d_{mean} (mean±error) (μm)	3.005±0.027	4.000±0.043	5.000±0.042	6.007±0.040	6.976±0.057	7.979±0.075	8.956±0.082	10.00±0.05	12.01±0.11
size distribution (μm)	0.029	0.04	0.05	0.06	0.07	0.09	0.09	0.09	0.12
coefficient of variation, CV (%)	1.1	1.0	1.0	1.0	1.0	1.1	1.0	0.9	1.0
nominal density ρ_{nom} (g/cm^3)	1.05 (polystyrene)								
catalog number	4203A	4204A	4205A	4206A	4207A	4208A	4209A	4210A	4212A
lot number	34742	203429	214115	204557	188878	36698	43716	36342	44085
vendor	Thermo Scientific Duke Standards								

nominal diameter d_{nom} (μm)	9.2
diameter d_{mean} (mean±error) (μm)	not available
size distribution (μm)	
coefficient of variation, CV (%)	4.8
nominal density ρ_{nom} (g/cm^3)	2.0 (silica)
catalog number	SiO2MS-2.0 9.2μm
lot number	171115-1048
vendor	Cospheric

Supplementary Table 2 | Manufacturer specifications for all particles used in this study.

Supplementary Notes

Supplementary Note 1: Theory

Here, we calculate the effect on the resonant frequency of a particle flowing through a suspended micro- or nanochannel resonator. The channel is assumed to have its axis aligned to the neutral axis of the resonator[1]. There are four natural length scales that arise: the velocity displacement amplitude, ϵ, the particle radius, a, the channel height, H, and the cantilever length L. All position variables are non-dimensionalized with respect to H unless otherwise specified; specifically, the lengthwise position coordinate x^* is scaled by L (but x is scaled by H) and the displacement function, $W(x^*)$, is scaled by ϵ. All other variables are non-dimensionalized with respect to the following scales; time, t, by $1/\omega$ where ω is the angular frequency of the resonator (in absence of the suspended particle), velocities by $\epsilon\omega$, and pressure by $\epsilon\omega\mu/H$ where μ is the shear viscosity of the fluid; this specifies the force and torque scales to be $\epsilon\omega\mu H$ and $\epsilon\omega\mu H^2$ respectively. All dependent variables have the exponential time dependence e^{-it}, e.g., $\sigma = \bar{\sigma}\mathrm{e}^{-it}$ where the real part of the expression gives the true (as measured) quantities; the superfluous overline is subsequently dropped from the analysis. A Taylor expansion of the base flow (i.e., in absence of the particle) around position x_0, gives[1],

$$\{\boldsymbol{u}_\mathrm{b}, p_\mathrm{b}\} = i\left\{-\left[\frac{H}{L}\frac{dW}{dx^*}\bigg|_{x_0^*}\left[\left(z - \frac{\sinh\lambda z}{\sinh\frac{\lambda}{2}}\right)\hat{\boldsymbol{x}} + (x - x_0)\hat{\boldsymbol{z}}\right] + W(x_0^*)\hat{\boldsymbol{z}}\right], \lambda^2 z\left(\frac{dW}{dx^*}\bigg|_{x_0^*}(x - x_0) + W(x_0^*)\right)\right\}, \tag{1}$$

where i is the imaginary unit, and $\lambda = (1 - i)\sqrt{\beta/2}$ where $\beta = \rho H^2\omega/\mu$ is the oscillatory Reynolds number and ρ is the fluid density.

Consider a rigid and spherical particle with radius a, and density ρ_p, located at position (x_0, z_0). This specifies two more non-dimensional parameters, a density ratio $\gamma = \rho_\mathrm{p}/\rho$ and a scaled radius, $R = a/H$ and $R^* = a/L$; throughout we assume that $R \ll 1$. This assumption allows us to linearize the flow field over the length scale of the particle, which, by using conservation of linear and angular momentum of the particle, specify its rigid body dynamics as,

$$\boldsymbol{u}_\mathrm{p} = U_x\hat{\boldsymbol{x}} + U_z\hat{\boldsymbol{z}} + \Omega_y\hat{\boldsymbol{y}} \times \boldsymbol{r}, \tag{2}$$

where $\hat{\boldsymbol{x}}$, $\hat{\boldsymbol{y}}$ and $\hat{\boldsymbol{z}}$ are the cartesian basis vectors, $\boldsymbol{r}$ is the position vector and

$$U_x = -iR^*\frac{dW}{dx^*}\bigg|_{x_0^*}\left(z - \frac{\sinh\lambda z_0}{\sinh\lambda/2}\right)\frac{1 + R\lambda + R^2\lambda^2/3}{1 + R\lambda + (2\gamma + 1)R^2\lambda^2/9}, \tag{3}$$

$$U_z = -iW(x_0^*)\frac{1 + R\lambda + R^2\lambda^2/3}{1 + R\lambda + (2\gamma + 1)R^2\lambda^2/9}, \tag{4}$$

$$\Omega_y = iR^*\frac{dW}{dx^*}\bigg|_{x_0^*}\frac{\lambda\cosh\lambda z_0}{2\sinh\lambda/2}\,\frac{1 + R\lambda + 6R^2\lambda^2/15 + R^3\lambda^3/15}{1 + R\lambda + (\gamma + 5)R^2\lambda^2/15 + \gamma R^3\lambda^3/15}. \tag{5}$$

As the particle performs the motion $\boldsymbol{u}_\mathrm{p}$, it generates a disturbance flow due to both its own motion, and the action of the base flow on the particle surface, via the no-slip and no-penetration boundary conditions. This disturbance flow is obtained using fundamental solutions[2], and is,

$$\{\boldsymbol{u}_\mathrm{d}, p_\mathrm{d}\} = \left[U_x + iR^*\frac{dW}{dx^*}\bigg|_{x_0^*}\left(z - \frac{\sinh\lambda z}{\sinh\lambda/2}\right)\right]\left\{\boldsymbol{u}_x^{(\mathrm{T})}, p_x^{(\mathrm{T})}\right\} + [U_z + iW(x_0^*)]\left\{\boldsymbol{u}_z^{(\mathrm{T})}, p_z^{(\mathrm{T})}\right\} + \left[\Omega_y - iR^*\frac{dW}{dx^*}\bigg|_{x_0^*}\frac{\lambda\cosh\lambda z_0}{2\sinh\lambda/2}\right]\left\{\boldsymbol{u}_y^{(\mathrm{R})}, 0\right\}$$
$$+iR^*\frac{dW}{dx^*}\bigg|_{x_0^*}\left(1 - \frac{\lambda\cosh\lambda z_0}{2\sinh\lambda/2}\right)\left\{\boldsymbol{u}^{(\mathrm{E})}, p^{(\mathrm{E})}\right\}. \tag{6}$$

The required fundamental solutions are given by

$$\left\{\boldsymbol{u}_i^{(\mathrm{T})}, p_i^{(\mathrm{T})}\right\} = \left(\frac{1}{4}(3 + 3R\lambda + R^2\lambda^2) - \frac{3 + 3R\lambda + R^2\lambda^2 - 3\mathrm{e}^{R\lambda}}{4R^2\lambda^2}\nabla^2\right)\left\{u_i^{(\mathrm{S})}, p_i^{(\mathrm{S})}\right\}, \qquad i \in \{x, z\}, \tag{7}$$

$$\left\{\boldsymbol{u}_x^{(\mathrm{S})}, p_x^{(\mathrm{S})}\right\} = \left\{A(r\lambda)\frac{\hat{\boldsymbol{x}}}{r} + B(r\lambda)\frac{x\boldsymbol{r}}{r^3}\right\} \qquad \left\{\boldsymbol{u}_z^{(\mathrm{S})}, p_z^{(\mathrm{S})}\right\} = \left\{A(r\lambda)\frac{\hat{\boldsymbol{z}}}{r} + B(r\lambda)\frac{z\boldsymbol{r}}{r^3}\right\}, \tag{8}$$

$$A(r\lambda) = 2e^{-r\lambda}\left(1 + \frac{1}{r\lambda} + \frac{1}{r^2\lambda^2}\right) - \frac{2}{r^2\lambda^2}, B(r\lambda) = -2e^{-r\lambda}\left(1 + \frac{3}{r\lambda} + \frac{3}{r^2\lambda^2}\right) + \frac{6}{r^2\lambda^2}, \tag{9}$$

$$\boldsymbol{u}^{(\mathrm{R})} = \frac{\mathrm{e}^{R\lambda}}{2(1 + R\lambda)}\left(\partial_z\boldsymbol{u}_x^{(\mathrm{S})} - \partial_x\boldsymbol{u}_z^{(\mathrm{S})}\right), \tag{10}$$

$$\left\{\boldsymbol{u}^{(\mathrm{E})}, p^{(\mathrm{E})}\right\} = \frac{1}{2}\left(-\frac{15 + 15R\lambda + 6R^2\lambda^2 + R^3\lambda^3}{9(1 + R\lambda)} + \frac{15 + 15R\lambda + 6R^2\lambda^2 + R^3\lambda^3 - 15\mathrm{e}^{R\lambda}}{9R^2\lambda^2(1 + R\lambda)}\nabla^2\right)\left(\partial_z\left\{u_x^{(\mathrm{S})}, p_x^{(\mathrm{S})}\right\} + \partial_x\left\{u_z^{(\mathrm{S})}, p_z^{(\mathrm{S})}\right\}\right), \tag{11}$$

where r is the radial spherical polar coordinate. The complete flow field within the SMR/SNR (under the assumption $R \ll 1$) is specified by $\boldsymbol{u} = \boldsymbol{u}_\mathrm{b} + \boldsymbol{u}_\mathrm{d}$ The disturbance flow therefore alters the force and torque exerted by the fluid on the cantilever; the change to the force and torque are calculated by,

$$F_\mathrm{d} = \hat{\boldsymbol{z}} \cdot \int_{-\infty}^{\infty}\int_{-\infty}^{\infty}(\boldsymbol{\sigma}_\mathrm{d}\cdot\boldsymbol{n})|_{z=z_0-1/2} + (\boldsymbol{\sigma}_\mathrm{d}\cdot\boldsymbol{n})|_{z=-z_0-1/2}\,dxdy, \tag{12}$$

$$M_\mathrm{d} = \hat{\boldsymbol{y}} \cdot \int_{-\infty}^{\infty}\int_{-\infty}^{\infty}\boldsymbol{r}\times(\boldsymbol{\sigma}_\mathrm{d}\cdot\boldsymbol{n})|_{z=z_0-1/2} + \boldsymbol{r}\times(\boldsymbol{\sigma}_\mathrm{d}\cdot\boldsymbol{n})|_{z=-z_0-1/2}\,dxdy, \tag{13}$$

where $\boldsymbol{\sigma}_\mathrm{d} \equiv -p_\mathrm{d}\boldsymbol{I} + \nabla\boldsymbol{u} + \nabla\boldsymbol{u}^\mathrm{T}$ is the usual stress tensor for an incompressible Newtonian fluid. The integration in Supplementary Eq. (12) can be performed analytically and gives,

$$F_\mathrm{d} = \frac{4\pi}{3}R^3\lambda^2(\gamma - 1)iW(x_0^*)\frac{1 + R\lambda + R^2\lambda^2/3}{1 + R\lambda + (2\gamma + 1)R^2\lambda^2/9}, \tag{14}$$

which is independent of the particle's height (i.e., z position) in the channel.

Unlike the force calculation above, the integration in Supplementary Eq. (13) for the torque must be performed numerically, however analytic expressions are obtained for small and large Reynolds numbers and are,

$$M_\mathrm{d} = -\frac{4\pi}{3}R^3 iR^*\frac{dW}{dx^*}\bigg|_{x_0^*}\begin{cases}\frac{2}{5}(\gamma - 1) + \frac{2}{5}\lambda^2 & \beta \ll 1\\ \frac{2}{3}\frac{15 + 15R\lambda + 6R^2\lambda^2 + R^3\lambda^3}{R^2\lambda^2(1 + R\lambda)} & \beta \gg 1\end{cases}. \tag{15}$$

Note that there is also a force exerted on the cantilever in the x-direction which has been neglected here because it produces a frequency shift response that is one order of magnitude smaller in the cantilever displacement amplitude than the frequency shift produced by F_{d} and M_{d}.

To calculate the effect on the resonant frequency due to the suspended particle, we examine the cantilever using Euler-Bernoulli beam theory. Under the assumption $H \ll L$, the applied force and torque act at a point along the length of the cantilever. The governing equations for the cantilever vibrations in the absence of a suspended particle, W_0, and in the presence of a suspended particle, W_1, are given by,

$$\frac{d^4W_i}{dx^{*4}} - \frac{L^3 m_{\mathrm{eff}}}{EI}\omega_i^2 W_i = 0, \qquad i \in \{0,1\} \qquad (16a,b)$$

where m_{eff} is the effective mass of the resonator, E its Young's modulus, I its second moment of inertia and ω_0 and ω_1 its resonant frequency in the absence of and presence of the suspended particle respectively. The usual clamped and free boundary conditions are applied,

$$W_i(0) = 0, \quad \left.\frac{dW_i}{dx^*}\right|_{x^*=0} = 0, \quad \left.\frac{d^2W_i}{dx^{*2}}\right|_{x^*=1} = 0, \quad \left.\frac{d^2W_i}{dx^{*2}}\right|_{x^*=1} = 0, \qquad i \in \{0,1\}. \qquad (17a,b)$$

Application of the point force and torque due to the suspended particle gives the additional boundary conditions,

$$\left.\frac{EI}{L^2}\frac{d^2W_1}{dx^{*2}}\right|_{x^*=x_0^{*-}} = -\mu\omega H^2 M_{\mathrm{d}}, \quad \left.\frac{EI}{L^3}\frac{d^3W_1}{dx^{*3}}\right|_{x^*=x_0^{*-}} = -\mu\omega H F_{\mathrm{d}}, \quad \left.\frac{d^2W_1}{dx^{*2}}\right|_{x^*=x_0^{*+}} = 0, \quad \left.\frac{d^3W_i}{dx^{*3}}\right|_{x^*=x_0^{*+}} = 0, \qquad (18)$$

where the $-$ and $+$ superscripts refer to approach from the left and right side of the particle position, x_0^* respectively. Multiplying Supplementary Eq. (16a) by W_1, Supplementary Eq. (16b) by W_0, taking the difference of the resulting expressions, and then integrating along the length of the cantilever leads to,

$$\Delta f = \Delta f_{\mathrm{disp}} + \Delta f_{\mathrm{rot}}, \qquad (19)$$

$$\Delta f_{\mathrm{disp}} = -\frac{f m_{\mathrm{b}}\, \alpha_{\mathrm{m}}\, W_0^2(x_0^*)}{2m_{\mathrm{eff}}}, \qquad \Delta f_{\mathrm{rot}} = -\, f\, \alpha_{\mathrm{v}}\, \mathcal{V} \left.\left(\frac{dW_0}{dx^*}\right)^2\right|_{x_0^*} \qquad (20a,b)$$

where $\Delta f = (\omega_1 - \omega_0)/2\pi$, m_{b} is the buoyant mass the particle and $\mathcal{V} = \rho V^{5/3}/\left(2\,[6\pi^2]^{1/3} m_{\mathrm{eff}}\, L^2\right)$ is the particle volume factor. The mass discrepancy and viscous enhancement factors are given respectively by

$$\alpha_{\mathrm{m}} = \Re\left[\frac{1 + R\lambda + R^2\lambda^2/3}{1 + R\lambda + (2\gamma + 1)R^2\lambda^2/9}\right], \qquad (21)$$

$$\alpha_{\mathrm{v}} = \Re\left[M^{(d)}\Big/\left(-iR^2\lambda^2\frac{4\pi}{3}R^*\left.\frac{dW}{dx^*}\right|_{x_0^*}\right)\right], \qquad (22)$$

where $\Re$ specifies the real part of the expression. Note that Supplementary Eq. (21) is identical to the corresponding expression in Ref. 3 in the limit of zero slip length. In general, α_{v} requires numeric evaluation and depends on the four parameters R, β, γ and z. In the limits of small and large Reynolds number, α_{v} reduces to

$$\alpha_{\mathrm{v}} = \begin{cases} \frac{3}{5}(\gamma - 1) + \frac{1}{20R^2}(1 - 12z^2)\sqrt{\beta} & \beta \ll 1 \\ \Re\left[\frac{15 + 15R\lambda + 6R^2\lambda^2 + R^3\lambda^3}{R^2\lambda^2(1 + R\lambda)}\right] & \beta \gg 1 \end{cases}; \qquad (23)$$

Supplementary Eq. (23) is used to plot the asymptotic limits in Fig. 2.

Influence of z-position on the viscous enhancement factor

The disturbance flow produced by the particle (and hence also the viscous enhancement factor) depends on the gradient of the base flow at the particle's location; the gradient of the base flow specifies the rotational and extensional flow components used in the analysis presented above. If the gradient of the base flow varies across the channel height, then, in general, the viscous enhancement factor also becomes dependent on the height of the particle within the channel (Supplementary Fig. 4). There are two key limits where the viscous enhancement factor becomes independent of z-position.

First, when $\beta \gg 1$, the base flow reduces to a purely extensional flow whose gradient is constant with respect to z; this can be seen by examining the $\beta \gg 1$ limit of Supplementary Eq. (1). Because the gradient of the base flow is constant with respect to z, the viscous enhancement factor, and therefore also the frequency shift, is independent of z; see the $\beta \gg 1$ limit of Supplementary Fig. 4 and Supplementary Eq. (23).

Second, when $\beta \ll 1$, the primary component of the base flow reduces to a purely rotational disturbance flow whose gradient is, again, constant with respect to z. If a non-neutrally buoyant particle ($\gamma \neq 1$) is suspended in this flow, then the dynamics are dominated by the primary flow (the secondary flow may be neglected here). Because the gradient of the base flow is constant with respect to z, so is the viscous enhancement factor and the frequency shift; see the $\beta \ll 1$ limits of Supplementary Fig. 4 and Supplementary Eq. (23), for particles where $\gamma \neq 1$.

In general, when the particle moves off axis there is also a contribution to the frequency shift due to its linear velocity in the x-direction. As discussed above, this contribution is neglected because it is order of magnitude smaller (in terms of the cantilevers displacement amplitude) than the frequency shifts described above.

Supplementary Note 2: Sources of experimental error

In our experiments, three main sources of error exist.

First, determination of V_{meas} from Δf^{node} involves a non-linear error propagation process, converting (Gaussian) frequency noise into a systematic error (Fig. 4b). Notably, the signal-to-noise ratio for Δf^{node} is smaller than Δf^{anti} (Supplementary Fig. 12). Consequently, particles of radii, $R < 0.25$, exhibit a variance in the signal, Δf^{node}, greater than that due to their nominal polydispersity (Supplementary Table 2). By performing Monte-Carlo simulations, we conclude that this enhancement is due to their lower signal-to-noise ratio (Supplementary Figs. 8, 9). When combining Δf^{node} with Δf^{anti} to calculate density, decreasing R decreases precision while not affecting accuracy (Supplementary Fig. 13c). Accuracy is not correlated with R, indicating that there is no systematic error in applying the theory.

Second, the derived theory does not account for the bounding walls of the channel. Therefore, we expect accuracy to decrease as the particle diameter approaches the channel height ($R \to 0.5$). This error depends on the ratio of the particle radius to both the viscous penetration depth, and the channel height. Even so, it is expected to be small because the disturbance flow decays rapidly from the particle, with a power-law between 2 and 4 in the radial distance (depending on βR^2). We experimentally observed that accuracy decreases slightly for $R > 0.4$, but is still greater than 99% (Supplementary Fig. 13c). Notably, we observed that for increasing R there is an additional uncertainty in defining the baseline frequency, f (Supplementary Fig. 11).

Third, analysis of experimental data implicitly assumes the particle is at the channel center ($z = 0$). This also leads to error because the measured signal, Δf^{node}, depends on the particle z-position (Supplementary Fig. 4). This error is negligible for $\beta \gg 1$ because the base flow is independent of z-position in this limit. As β decreases, the (unknown) variation in z-position can influence the experimental fitting. To quantify this error, we fit the experimental data to the theory where α_{v} as a function of z takes on its minimum and maximum values which correspond to the maximum and minimum extracted radius and density for each individual particle measurement (Supplementary Fig. 7). Quantifying this error with 95% confidence intervals over the entire experiment on each batch of particles (Supplementary Table 2), we find that for the devices with the four largest values of β, the error in density is negligible while the error in radius is always less than 10% (regardless of the unknown z-position). For the devices with the two smallest values of β, the uncertainty in the radius increases while that in the density remains small. Note that these bands give a worst-case scenario for the unknown z position. Due to the stochastic distribution of particle positions discussed below, we expect the true uncertainty to be much smaller.

We find that particle motion due to inertial migration within the cantilever is negligible. The channel length required for focusing of particles to their equilibrium positions can be approximated by $L = \pi\mu H^2/\rho U_{\text{m}} a^2 f_{\text{l}}$ where μ is the fluid's shear viscosity, H is the channel height, ρ is the fluid's density, U_{m} is the velocity at the center of the channel, a is the particle diameter and f_{l} is the lift coefficient (generally between 0.01 and 0.1)[4]. Using the transit times of the particles through the device (which are no faster than 0.1s) we approximate the maximum flow velocity to be $U_{\text{m}} < 0.007\,\text{m/s}$. Taking the remaining experimental parameters from Supplementary Tables 1 and 2 we find that the minimum channel length across any of the devices needed for significant inertial migration is 0.02 m, which is two orders of magnitude larger than the longest SMR used in this study. Therefore, particles will remain on the same streamline upon which that they enter the channel. Individual streamlines will be selected stochastically by different particles, and we therefore do not expect there to be significant bias towards any channel positions.

Effect of off-axis channel placement on the viscous enhancement factor

Due to imperfections in the manufacturing process, the fluid-filled cantilevers may not have their channels aligned perfectly to their neutral axis. When this happens, a flow is generated which is pumped in and out of the channel as it is lengthened and shortened due to the cantilever's vibrations[1]. Because the fluid acoustic wavelengths for the cantilever vibrations for all devices considered in this study are much larger than the cantilever lengths, it is sufficient to consider incompressible flow. For high inertia, $\beta \gg 1$, i.e., where the rotation signal, Δf_{rot}, is independent of z-position, the base flow is a plug flow which acts to vibrate the particle tangentially to the length of the cantilever. This induces a force on the cantilever tangential to its length, i.e., negligible in the small amplitude limit considered in this study (there will be a change in frequency at the order of vibration amplitude squared which may be calculated through use of higher order beam theories). For finite β, i.e., where the effects of vorticity are significant throughout the interior of the cantilever, off axis placement can potentially play a role in the viscous enhancement factor due to rotation of the suspended particle. However, for the present mode 2 measurements in the cantilevers used in this study this effect is small for two reasons: (i) The node is close to the end of the cantilever where the pump flow is weakest (reducing any off-axis effect by an order of magnitude). (ii) The height of the channel is always much larger than the thickness of the top and bottom lids ($H > H_{\text{top}}, H_{\text{bot}}$, Supplementary Table 1) and so any offset of the channel from the neutral axis must also be small (i.e., it will be proportional to the difference in thickness between the top and bottom lids); this multiplies any off-axis effect by at most an $O(1)$ number. Combining (i) and (ii) we see that any frequency shift due to off-axis effects is at least two orders-of-magnitude smaller than the frequency shift studied here due to the rotating walls.

Supplementary References

[1]Sader, J. E., Burg, T. P. & Manalis, S. R. Energy dissipation in microfluidic beam resonators. *J. Fluid Mech.* **650**, 215–250 (2010).

[2]Pozrikidis, C. A singularity method for unsteady linearized flow. *Phys. Fluids A* **1**, 1508–1520 (1989).

[3]Collis, J. F., Olcum, S., Chakraborty, D., Manalis, S. R. & Sader, J. E. Measurement of Navier slip on individual nanoparticles in liquid. *Nano Lett.* https://doi.org/10.1021/acs.nanolett.1c00603 (2021).

[4]Di Carlo, D. Inertial microfluidics. *Lab Chip* **9**, 3038–3046 (2009).
